# Metabolic effects of prolactin

**DOI:** 10.3389/fendo.2022.1015520

**Published:** 2022-09-27

**Authors:** Rosa Pirchio, Chiara Graziadio, Annamaria Colao, Rosario Pivonello, Renata S. Auriemma

**Affiliations:** ^1^ Dipartimento di Medicina Clinica e Chirurgia, Sezione di Endocrinologia, University of Naples Federico II, Naples, Italy; ^2^ Unesco Chair for Health Education and Sustainable Development, “Federico II” University, Naples, Italy

**Keywords:** metabolism, glucose profile, insulin metabolism, lipid profile, hyperprolactinemia, hypoprolactinemia, HomeoFIT-PRL

## Abstract

Over the last years, the metabolic role of PRL has emerged. PRL excess is known to promote weight gain, obesity, metabolic syndrome, and impairment in gluco-insulinemic and lipid profiles, likely due to the suppression of physiologic dopaminergic tone. Prolactin receptors and dopamine receptors type 2 have been demonstrated to be expressed on both human pancreatic β- cell and adipocytes, supporting a key role of prolactin and dopamine in peripheral metabolic regulation. Medical treatment with the dopamine agonists bromocriptine and cabergoline has been demonstrated to decrease the prevalence of metabolic syndrome and obesity, and significantly improve gluco-insulinemic and lipid profiles. In hyperprolactinemic men with concomitant hypogonadism, correction of hyperprolactinaemia and testosterone replacement has been proven to restore metabolic impairment. In turn, low prolactin levels have also been demonstrated to exert a detrimental effect on weight gain, glucose and lipid metabolism, thus leading to an increased prevalence of metabolic syndrome. Therefore, PRL values ranging from 25 to 100 mg/L, in absence of other recognizable pathological causes, have been proposed to represent a physiological response to the request for an increase in metabolic activity, and nowadays classify the so-called HomeoFIT- PRL as a promoter of metabolic homeostasis. The current review focuses mainly on the effects of hyperprolactinemia and its control by medical treatment with DAs on the modulation of food intake, body weight, gluco-insulinemic and lipid profile. Furthermore, it provides the latest knowledge about the metabolic impact of hypoprolactinemia.

## Introduction

Beyond the classical well-known effects on gonadal function, reproduction, and lactation, prolactin (PRL) has recently been identified to exert an intriguing metabolic role (1, 2).

Many physiological and pathological conditions could result in PRL excess. Indeed, hyperprolactinemia could be determined not only by PRL-secreting pituitary adenomas, but also by numerous other conditions involving physiological status such as pregnancy and lactation, systemic disorders like chronic renal failure and cirrhosis or several pharmacological treatments (3). Among non-pathological causes of hyperprolactinemia the hook effect and macroprolactin are the most common. The hook effect is responsible for the discrepancy between tumor size and PRL levels, which generally requires a further diagnostic step based on serum sample dilution (3). Macroprolactin is an isoform of PRL with a greater molecular weight but reduced biological activity, occurring in about 20% of cases and associated with asymptomatic hyperprolactinemia. Both conditions lead to misdiagnosis, unnecessary investigation, and inappropriate treatment of hyperprolactinemic patients.

Regardless of the nature of hyperprolactinemia (4–7), PRL excess is known to influence the orexigenic–anorexigenic systems that regulate appetite determining hyperphagia and increase in food intake, leading to weight gain till to overt obesity (8–10). Consequently, metabolic disorders are common findings in patients with PRL excess and include metabolic syndrome and related disorders in gluco-insulinemic and lipid profile. Noteworthy, PRL deficiency, defined as PRL levels ≤ 7 µg/L, has been recently reported to impair metabolic homeostasis to a similar extent as compared to PRL excess (11).

Hyperprolactinemia has been associated with alterations in gluco-insulinemic metabolism (2, 12–17). In particular, a reduction in glucose tolerance and a raise in fasting insulin (FI) have been found in patients with hyperprolactinemia (18–20), independently from Body Mass Index (BMI). As a consequence, impaired FI-derived index has also been encountered in hyperprolactinemic patients as compared to healthy controls, particularly decreased insulin sensitivity index (ISI, 21) and increased homeostatic model assessment index (HOMA-IR, 21-23). Conversely, the impact of hyperprolactinemia on lipid metabolism seems to be less clear since no univocal results have been reported so far. Indeed, in previous studies both inhibition of lipogenesis (8) and of release in adipokines (10, 21–24) and the promotion of lipogenesis (5, 25) and of central leptin resistance (26, 27) have been observed together with favorable (28) or unfavorable (8) effect on lipid storage, thus suggesting complex regulatory mechanisms behind these effects that need further clarifications.

Restoration of normal PRL levels through proper therapy has been demonstrated to improve metabolic impairment. Dopamine-agonists (DAs), mainly bromocriptine (BRC) and cabergoline (CAB), represent the treatment of choice for patients with hyperprolactinemia (29–31). Metabolic benefits of DAs have been demonstrated to occur even in absence of PRL excess. In diabetic non-hyperprolactinemic patients, significant improvement in glucose profile has been reported using both drugs, regardless of concomitant hyperprolactinemia (32–40). Particularly, a fast-absorbing form of BRC, namely bromocriptine-Quick Release, has been shown to reduce plasma glucose levels in obese nondiabetic hyperinsulinemic women on a weight-maintaining diet (35), and to induce improvement of glucose tolerance and reduction in weight in both diabetic and nondiabetic subjects (34, 38). For these reasons, bromocriptine-Quick Release has been officially approved as an additional treatment for type 2 diabetes mellitus (DM) in the US (38). Noteworthy, a significant decrease in glycated hemoglobin (HbA_1c_) levels has been reported after the addition of CAB at the dose of 0.5 mg/week to previous glucose-lowering therapy, 65% of these patients achieving HbA_1c_ <7% as compared to 45% of controls after 3 months of treatment (40).

In patients with prolactinomas, BRC has been reported to induce a significant decrease in body weight and per cent body fat (41–48), together with a notable improvement in glucose homeostasis and insulin resistance (6, 7, 41, 42). In obese men with prolactinomas, 6-month treatment with either BRC or CAB has been demonstrated to determine a significant reduction in body weight (43). Similarly, a significantly lower body fat percentage has been reported in patients administered with CAB treatment as compared to newly diagnosed treatment-naïve patients (44, 45). Therefore, these results suggest that PRL normalization induced by CAB could reduce body fat percentage and the concomitant risk of obesity and related metabolic implications. As expected, long treatment with DAs, mainly CAB, has been demonstrated to significantly improve both gluco-insulinemic and lipid metabolism (45–48). CAB reportedly exerts a direct action on metabolic homeostasis since the improvement in metabolism seen while on CAB has been demonstrated to be directly related to the drug dose, rather than to be dependent on the degree of PRL reduction (47, 48).

Furthermore, in male patients with hyperprolactinemia, complete recovery of gonadal function and testosterone deficiency following DAs therapy occurs in about 50% of cases, but permanent hypogonadism may persist in half of the patients (49–51). In these persistent hypogonadal patients, complete recovery from impaired metabolism has been reported only after the addition of testosterone to CAB (52), particularly concerning insulin resistance and metabolic syndrome (MetS).

**Figure 1:**
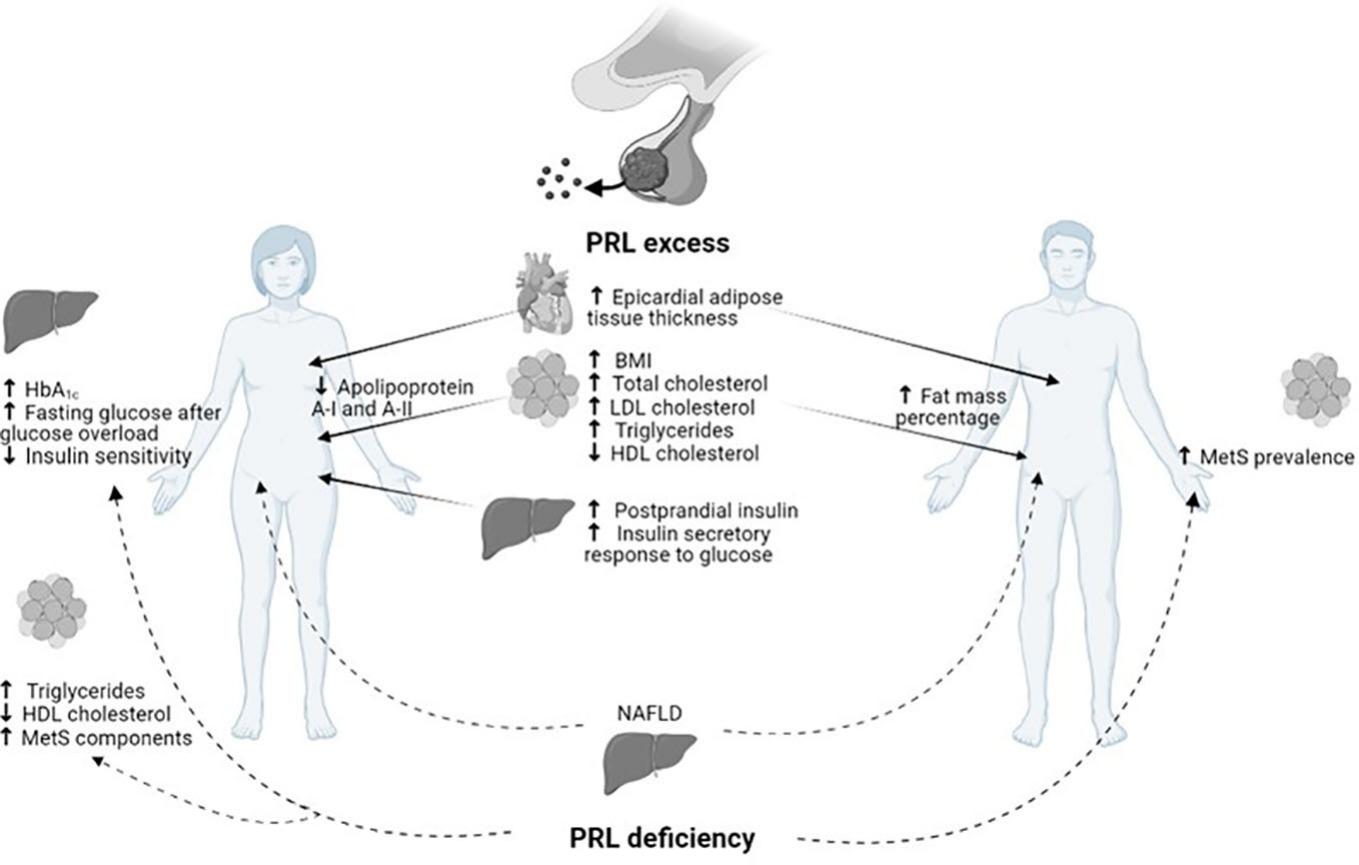
Gender related metabolic impact of PRL excess and deficiency.

On the other hand, the detrimental metabolic effect of hypoprolactinemia, defined as PRL ≤7 µg/L, has recently emerged (11). The definition of hypoprolactinemia first appeared in 2009, when a study on male patients with sexual dysfunction detected a higher risk of MetS in patients with PRL levels <5 µg/L, particularly middle-aged and elderly men (53, 54). Subsequently, several studies have been conducted in order to clarify the metabolic impact of hypoprolactinemia; unexpectedly the association between low PRL levels and insulin resistance, DM, and MetS was discovered (11), thus suggesting the need to achieve PRL levels within the normal range to ensure metabolic homeostasis (**Figure 1**). As shown in **Figure 1**, besides several metabolic effects common to women and men, both PRL excess and deficiency might further result in some peculiar metabolic actions according to gender. Particularly, a reduction in apolipoprotein A-I and A-II has been demonstrated in hyperprolactinemic women, whereas an increase in body fat mass percentage has been revealed only in men with hyperprolactinemia. Similarly, the detrimental impact of PRL deficiency on glucose metabolism and adipose tissue function has been shown only in women.

The current review focuses mainly on the effects of hyperprolactinemia and its control by medical treatment with DAs on the modulation of food intake, body weight, gluco-insulinemic and lipid profile. Furthermore, it provides the latest knowledge about the metabolic impact of hypoprolactinemia.

### Effects on food intake and body weight

Chronic PRL excess has been associated with increased food intake and weight gain, leading to obesity (6–10). In hyperprolactinemic patients, the raise in appetite has been mainly ascribed to the functional block of dopaminergic tone induced by hyperprolactinemia. Physiologically, dopaminergic tone exerts a key role in reducing food intake and promoting energy expenditure (4–7, 55), supporting the hypothesis of dopamine involvement in body weight regulation. Preclinical studies using genetically modified hyperprolactinemic mice have revealed that chronic PRL excess is able to modulate the expression of genes involved in the orexigenic-anorexigenic balancing at the hypothalamic level. Particularly, after the development of hyperprolactinemia-induced obesity in these mice, a significant increase in mRNA levels of the orexigenic hormones agouti-related peptide (AGRP) and neuropeptide Y (NPY) were observed in the hypothalamic arcuate and dorsomedial nuclei, respectively (56). Likewise, dopaminergic tone suppression, together with the raise in hypothalamic levels of the appetite-stimulating hormones NPY and corticotrophin-releasing hormone (CRH, 8–10) have been identified as potential mechanisms determining hyperphagia and consequent weight gain in patients with hyperprolactinemia. Indeed, in patients newly diagnosed with prolactinoma, BMI has been reported to be significantly higher than controls, although no significant difference between the two groups in fat mass percentage, measured by dual-energy X-ray absorptiometry (DXA) at six sites (arms, legs, trunk, android, gynoid, total body), has been detected. Otherwise, analyzing these patients according to gender, a significantly higher fat mass percentage at all sites was found in male patients as compared to controls, whereas no difference in fat mass was observed when comparing female patients and controls (57). Consistent with these results, patients with prolactinoma seem to have a tendency to store more fat than healthy controls, even in unconventional sites. Indeed, epicardial adipose tissue thickness, considered a surrogate marker of visceral fat and a novel cardiovascular risk indicator, has been recently discovered to be higher in patients with prolactinoma as compared to healthy controls, even if similar cardiac systolic and diastolic functions were observed between the two groups (58). In a recent study focusing on the metabolic impact of pituitary adenomas, patients with prolactinoma have been confirmed to show significantly higher BMI and waist circumference as compared to healthy controls, whereas no significant anthropometric difference between patients with prolactinoma and other pituitary adenomas was found, neither non-functioning nor secreting other pituitary tropins (59). These findings led to the conclusion that obesity is very common in presence of PRL excess, raising the question of whether the achievement of PRL control by DAs or surgery can restore metabolic impairment. In fact, several studies have documented the decrease in body weight and BMI following PRL normalization in patients treated with DAs (5, 60). In hyperprolactinemic patients, the activation of dopamine receptor type 2 (D2DR) has been suggested as a potential mechanism through which the decrease in BMI and body fat percentage is achieved after PRL normalization induced by CAB (44). Particularly, beyond the reduction in BMI, also waist circumference has been found to be significantly reduced after long-term treatment (up to 5 years) with CAB (47, 48, 52), thus suggesting a decrease in visceral obesity, which is known to have a pivotal role in inflammation and cardiovascular risk. The investigation of the metabolic impact of pituitary surgery, as a therapeutic strategy alternative to DAs, has provided controversial results. Consistent with the crucial role of PRL normalization in triggering the achievement of weight reduction in patients with prolactinomas, a study investigating changes in BMI long-term (about 52 months) after first-line surgery has revealed a significantly greater decrease in BMI in patients who underwent pituitary surgery as compared to those administered with DAs, the prevalence of PRL normalization being slightly higher in the first group (61). In contrast, a study investigating the impact of surgery and high-dose CAB (≥ 2 mg/week) in patients with prolactinomas resistant to conventional CAB doses documented a neutral effect of pituitary surgery on body weight and BMI (62), raising the hypothesis that a relevant effect on body weight and BMI is strongly dependent on PRL normalization and that in patients with long-term uncontrolled hyperprolactinemia by DAs the beneficial impact of surgery on body composition may be limited.

In males affected by prolactinomas, hypogonadotropic hypogonadism is a common finding; noteworthy low testosterone levels might further influence weight gain besides PRL excess in these patients, as body weight, BMI, and waist circumference have been found to be significantly higher as compared to non-hypogonadal patients after treatment with CAB and testosterone replacement (52). Similarly, a two-fold higher risk of suffering from obesity and MetS has been encountered in men with testosterone and dihydrotestosterone levels in the lower quartiles (63). On the other hand, testosterone levels could also be influenced by body weight since a 5% reduction in body weight *per se* has been demonstrated to significantly increase testosterone levels, at least in ageing men (64), with a greater improvement being achievable by further weight loss. Altogether this evidence leads to the hypothesis that in males with hyperprolactinemia-induced hypogonadism visceral obesity might be widely influenced by androgen deficiency, therefore weight loss might be ascribed not only to a beneficial impact of CAB treatment but also to proper testosterone replacement (52).

Moreover, the detrimental effect of PRL excess on BMI has also been demonstrated to be a common finding in young patients with prolactinoma onset during childhood since a prevalence of obesity of about 35% has been reported at diagnosis at this age (65, 66). Of note, bearing a prolactinoma in childhood could negatively affect metabolic health even during adulthood since obesity has been demonstrated to persist following treatment (65, 66), even when the patient is cured (66).

Hyperprolactinemia results in impaired body composition with greater waist circumference, body weight and BMI, and increased body fat percentages only in men. An improvement in body weight and BMI is possible by normalizing PRL levels, although after long-term therapy. Furthermore, in hyperprolactinemic men with concomitant androgen deficiency, the addition of testosterone replacement to DAs is mandatory to reverse the effects of both on body composition.

Otherwise, PRL deficiency has been found associated with impaired body composition. Data in adults are still lacking. In obese non-hyperprolactinemic children, PRL levels have been found to be lower as compared to normal weight children, PRL being inversely correlated to BMI, HOMA-IR, and Interleukin-6 (IL-6) levels and directly to HDL levels. In these obese subjects, PRL has been identified as a prognostic marker able to predict MetS development with higher sensitivity and specificity than pro-inflammatory cytokines IL-6 and Tumor Necrosis Factor-α. After 12 months of lifestyle changes, an improvement in BMI, insulin, and lipid profile was observed together with the increase in PRL levels, although still within the lower quartile (mean PRL <6 µg/L, 72).

### Effects on metabolic syndrome

PRL excess is known to promote MetS in approximately one-third of patients, ranging from 23–50% in the different series (1). The impact of DAs treatment on MetS prevalence has been investigated in a few studies (45, 47, 48, 52). A reduction in MetS prevalence of approximately 5% has been reported after 6-60 months of therapy with DAs, mainly using CAB (48). Obviously, this result might be ascribable to the concomitant weight loss, and improvement in gluco-insulinemic and lipid metabolism induced by DAs therapy. Particularly, DAs seem to exert a direct ameliorative effect on pancreatic β-cell and adipocyte functions, thus suggesting that these drugs could be useful as adding-on therapy in normoprolactinaemic unhealthy obese failing to achieve optimal metabolic control despite standard therapies.

Conversely, a higher prevalence of MetS was observed in middle-aged and elderly male patients with sexual dysfunction and PRL levels <5 µg/L (54). Particularly, PRL levels were demonstrated to be inversely correlated to MetS prevalence in these men (54). Similarly, a longitudinal analysis in healthy subjects has revealed a significant trend toward lower PRL concentrations with an increasing number of MetS components in women, although no association between PRL and MetS incidence has been shown in multivariable regression models (67). Interestingly, similar results have not been confirmed in men (67).

### Effects on gluco-insulinemic profile

Several studies have focused on the association between PRL excess and impairment in gluco-insulinemic profile (68).

PRL receptors (PRLR) expression has been demonstrated on insulin-secreting cell lines (13) and rat β-cells (14), the expression being reported to be increased during pregnancy (15). Indeed, pregnancy represents a model of prolonged exposure to hyperprolactinemia that allows studying mechanisms through which PRL could affect gluco-insulinemic metabolism. Peculiar PRL-induced changes in pancreatic β-cells mass and function have been demonstrated in rodents during pregnancy (12–17). A concomitant increase in PRL levels, β-cell mass and glucose-induced insulin hypersecretion has been shown to occur in islets of Langerhans in adaptation to gestation (12). Similarly, in *in vitro* studies the exposition of isolated pancreatic rat islets to PRL has been demonstrated to result in a raise in insulin secretion together with β-cell proliferation (13, 16). Moreover, PRL overexpression in β-cells has been observed to induce inappropriately elevated serum insulin concentrations, increased islet insulin content, and sustained β-cell replication (17). Either in rats or in humans, PRL has been revealed to promote β-cell proliferation, insulin gene transcription, and glucose-dependent insulin secretion (69–72). Consistently, chronic hyperprolactinemia has been associated with impaired insulin secretion, characterized by postprandial hyperinsulinemia and exceeding insulin secretory response to glucose in humans (73–75).

Consistent with PRL detrimental effect on pancreatic β-cell function, the restoration of PRL levels within normal ranges reportedly improved the gluco-insulinemic profile. D2DR has been found on pancreatic β-cells, thus a direct role of DAs on insulin secretion and cell proliferation has been hypothesized. Indeed, treatment with the selective D2DR agonist quinpirole has been found to inhibit glucose-stimulated insulin secretion (76). Similarly, in rodents, insulin secretory response is inhibited by a single injection with the dopamine precursor L-dopa (77, 78), whereas reduced insulin secretion upon a glucose load has been reported in humans with Parkinson’s disease treated with L-dopa (79). Likewise, a significant amelioration in glucose profile and insulin resistance after 6-months of treatment with either BRC or CAB has been reported in patients with prolactinoma (44, 45, 47, 48, 52). FI and HOMA-IR have been reported to significantly reduce following long-term CAB therapy, particularly in patients receiving CAB doses higher than 0.5 mg/week (47, 48), being independent of changes in body weight and BMI (48). In addition, insulin-derived indices HOMA-β and ISI have been found improved compared to baseline after CAB treatment (48). Although this effect could be explained at least partly by weight loss and lifestyle changes during therapy with CAB (80, 81), the assumption of CAB beneficial direct effect on insulin secretion and sensitivity could not be excluded. CAB dose has been demonstrated to be the best predictor of a per cent decrease in FI (48), thus supporting the hypothesis of a direct beneficial effect of CAB itself on pancreatic β-cell function. Furthermore, insulin sensitivity amelioration has been found to be associated with a significant reduction in glucose levels either in lean or obese patients, regardless of PRL levels (48). In hyperprolactinemic male patients with concomitant hypogonadotropic hypogonadism, FI, HOMA-IR, HOMA-β, and ISI have been reported to significantly improve after long-term therapy with CAB, being further ameliorated by androgen replacement and consequent testosterone normalization (52). Interestingly, CAB dose has been shown to significantly correlate with FI and HOMA-β in these patients (52), further strengthening the hypothesis of a direct dopaminergic role in the regulation of insulin secretion (48). Additionally, testosterone levels have been found to significantly correlate with ISI (52), suggesting a potential direct beneficial action of replacement therapy on the regulation of insulin sensitivity. Supporting the role of PRL normalization on glucose metabolism amelioration, fasting glucose (FG) was found to be significant decreased in both patients undergone pituitary surgery and those administered with DAs as first-line treatment for prolactinoma after long-term follow-up (61). Recently, the impact on the gluco-insulinemic profile of different therapeutic approaches has also been investigated in patients affected by prolactinomas resistant to CAB conventional dose therapy to determine whether high-dose CAB therapy (i.e., ≥ 2 mg/week) and pituitary surgery could exert a similar metabolic impact in these patients (62). After 12 months of treatment, a significant improvement in FG, FI, HOMA-β, and ISI were found in patients who were administered high-dose CAB whereas no significant change was found in those undergone pituitary surgery despite a greater degree of PRL reduction after the latter treatment, thus suggesting that the gluco-insulinemic metabolic improvement is mainly ascribable to CAB direct effect rather than the entity of PRL variation. In patients treated with pituitary surgery, per cent change in FG 12 months after neurosurgery was significantly correlated not only to per cent change in PRL but also to CAB dose received after neurosurgery in those patients who resulted not cured by surgical treatment, confirming the key direct role of CAB. A further potential mechanism involved in insulin sensitivity improvement might be the impact of D2DR activation on Growth Hormone (GH) levels and/or in Insulin-like Growth Factor- 1 (IGF-I) bioactivity since in healthy non-obese subjects the activation of D2DR has been shown to influence circulating GH and IGF-I levels (82), IGF-I bioactivity reportedly being inversely associated to insulin sensitivity (83).

Therefore, PRL excess is associated with postprandial hyperinsulinemia and exceeding insulin secretory response to glucose in humans; improvement in FG, FI and related indices is achieved during DAs therapy.

A significant amelioration in glucose profile induced by either BRC or CAB has been documented even in diabetic non-hyperprolactinemic patients (34–40). Particularly, bromocriptine-Quick Release has been found to decrease plasma glucose levels and improve glucose tolerance in both diabetic and nondiabetic subjects (34, 35, 38). Likewise, in a recent double-blind placebo-controlled study in diabetic normoprolactinaemic patients with DM in suboptimal control (HbA_1c_ ranging 7–10%), the addition of CAB at the dose of 0.5 mg/week to the usual treatment by ≥2 oral glucose-lowering drugs has been demonstrated to significantly decrease HbA_1c_ levels after 3 months as compared to placebo (40). Particularly, HbA_1c_ level <7% was achieved in 65% of patients as compared to 45% of controls (40). Molecular mechanisms through which BRC exerts this glucose-lowering effect have been not clearly elucidated yet. Recently, a preclinical study on skeletal muscle of diabetic rats tried to clarify these mechanisms, highlighting the multiple molecular targets of BRC (84). In fact, BRC has been shown to promote insulin sensitivity in the skeletal muscle cell by reducing the inflammatory signal (IL-6/JAK2/p-STAT3/SOCS3) and enhancing the PPAR- γ/adiponectin signaling, involved in the storage of fatty acids and glucose metabolism, leading to the activation of the insulin signaling pathway (p-IR/p-AKT/GLUT4, 90). Furthermore, BRC has been demonstrated to improve leptin and GLP-1 levels, suggesting that BRC may exert a wide action on glucose regulation also through the regulation of food intake and endocrine pancreatic secretion, respectively (84).

As for body composition and BMI, a detrimental effect of PRL deficiency has been documented also on gluco-insulinemic metabolism. In large population cohorts of non-hyperprolactinemic subjects, lower PRL levels have been found linked with glucose impairment (67, 85). Particularly, low PRL levels have been found associated with DM in age- and multivariable-adjusted regression models, although after a median follow-up of 5 years no relation between PRL values and incident DM has been revealed (67), as well as no direct correlation between FG and PRL levels has been noted (86). Data of men and postmenopausal women were analyzed in another cohort study, revealing that subjects with impairment in glucose metabolism (impaired fasting glucose and impaired glucose tolerance) or overt DM showed lower circulating PRL levels as compared to subjects with normal glucose tolerance. Moreover, the risk for glucose metabolism and DM impairment decreased across PRL quartiles in multinomial logistic regression analyses. Only in women, lower levels of fasting and postprandial glucose, HbA_1c_, and HOMA-IR, lower prevalence of DM family history, and higher HOMA-β have been observed in those belonging to the highest PRL quartile (85). Likewise, women belonging to the highest PRL quartile had a lower likelihood to develop DM compared with those in the lowest quartile after a mean follow-up of about 4 years, whereas this trend was not observed in men (87). The relationship between circulating PRL concentrations and DM risk has been also studied in the large cohort taking part to Nurses’ Health Study (NHS) and NHS-II with up to 22 years of follow-up. In this study, plasmatic PRL has been found to be inversely associated with DM risk in both genders (87). Lower serum PRL levels have also been identified as a negative predictive factor of a higher risk to develop impaired fasting glucose and DM after childbirth (88). In order to clarify the effect of low PRL levels during CAB treatment on gluco-insulinemic profile, a comparison between young women under CAB therapy and healthy controls has been recently carried out (89). Higher HbA_1c_ and FG levels after glucose overload and lower insulin sensitivity were seen in the former than in the latter. A decrease in CAB dose resulted in an increase in PRL levels and an improvement of gluco-insulinemic impairment (89).

### Effects on lipid profile

Impairment in lipid profile is a common finding in patients with PRL excess. As previously reported, newly diagnosed treatment-naïve hyperprolactinemic patients have been demonstrated to have higher body fat percentages as compared to controls (43, 89). Particularly increased total, LDL-cholesterol and triglycerides, and decreased HDL-cholesterol have been observed more frequently in patients with prolactinomas as compared to healthy control subjects (41, 90–93). Therefore, a direct correlation between PRL levels and lipid fractions has been hypothesized (42, 91). Moreover, a significant decrease in apolipoprotein A-I and A-II has been found in hyperprolactinemic women as compared to healthy control women (94); among these women with hyperprolactinemia, a significant decrease in apolipoprotein B has also been observed in those with high estradiol levels as compared to those with low estradiol levels (94). Impairment in apolipoproteins biosynthesis induced by PRL excess has been proposed as potential mechanism responsible for lower apolipoproteins levels in these patients; in addiction a decrease in apolipoproteins degradation due to liver function alteration has been hypothesized in the subgroup with high estradiol levels (94).

A direct effect of PRL on lipid metabolism has been supposed since an increase in PRL receptors expression has been found during adipocyte differentiation, suggesting their involvement in mature adipocyte regulation (95–98). Moreover, the dopaminergic tone might also have an impact on adipose tissue regulation due to the expression of D2DR on human adipocytes (99). Likewise, PRL expression and release have been demonstrated to be inhibited by DAs in adipocytes in *in vitro* studies. That DAs may exert a beneficial impact on adipose tissue function in patients with hyperprolactinemia has also been supported by several clinical studies. Visceral adiposity index, a surrogate parameter of adipose tissue dysfunction associated with cardiometabolic risk in healthy subjects, significantly decreased after CAB long-term therapy in hyperprolactinemic patients (100), particularly using doses higher than 0.5 mg/week (47, 48), even apart from the extent of PRL reduction (48). In patients with PRL excess, either BRC or CAB have been demonstrated to improve lipid profile regardless of changes in body weight and BMI (39, 41, 44, 47, 48, 52, 101), suggesting that DAs might have a direct beneficial impact on lipid metabolism. Indeed, DAs could influence lipid metabolism through D2DR activation independently of food intake and body weight (32, 34, 36, 102). Of note, a significant decrease in total, LDL-cholesterol and in triglycerides has been revealed following CAB therapy in male hyperprolactinemic patients with concomitant hypogonadism, with no further improvement in lipid profile after 12 months of testosterone replacement therapy (52). Based on these findings, amelioration in lipid profile might be not only due to PRL normalization and subsequent decrease in BMI but mainly ascribable to DAs direct effect (48, 52). Otherwise, few pieces of evidence about a substantially neutral effect of dopamine and/or DAs on lipid profiles are available (60, 103). Possibly, the small size of patient cohorts, the heterogeneity of administered treatments, and the short-term treatment with DAs might explain these not univocal results.

Diverse first-line therapeutic approaches seem to differently affect lipid metabolism since a significant reduction in triglycerides has been found in patients undergone pituitary surgery whereas a significant decrease in total cholesterol has been shown after DAs therapy (62). Similarly, the role of dopaminergic activation in adipocyte function modulation has also been highlighted in patients with prolactinomas undergone pituitary surgery due to resistance to CAB conventional therapy. Although in these patients a significant decrease in total cholesterol and triglycerides has been found 12 months after pituitary surgery, at the regression analysis CAB dose has been demonstrated to be the best predictor of per cent decrease in LDL in patients not cured after neurosurgery and thus requiring medical treatment (63).

Therefore, the increased levels of PRL result in an impairment of apolipoprotein biosynthesis, increased total, LDL-cholesterol and triglycerides, and decreased HDL-cholesterol together with higher body fat percentages in hyperprolactinemic patients as compared to controls. Restoration of PRL levels within the normal ranges allows to improve lipid metabolism, particularly CAB ameliorates lipid profile regardless of changes in body weight and per cent change in PRL.

Few data are nowadays available on the impact of PRL deficiency on lipid profile. Low PRL levels have been found associated with higher triglycerides and lower HDL-cholesterol levels in young hyperprolactinemic women during CAB therapy as compared to healthy controls, whereas no significant difference in lipid profile was found when comparing young women administered with CAB achieving normal PRL levels and healthy controls. Furthermore, in the former group the improvement of lipid fractions has been achieved by reducing the CAB dose (89).

PRL may have a role also in intrahepatic lipid storage since circulating PRL has been demonstrated to be lower in patients with non-alcoholic fatty liver disease (NAFLD) compared to healthy controls (104). A further aim of this research was the evaluation of the expression of the gene encoding the PRLR and signaling molecules involved in hepatic lipid metabolism in both human liver and HepG2 cells, a human cell line derived from hepatocellular carcinoma. Hepatic PRLR gene expression was significantly reduced in patients with NAFLD and negatively correlated with CD36 gene expression, a key transporter of free fatty acid uptake in the liver involved in NAFLD development. PRL levels were significantly lower in patients with severe hepatic steatosis as compared to those with mild-to-moderate hepatic steatosis in both sexes. In free fatty acid-induced HepG2 cells, PRL treatment or PRLR overexpression significantly reduced the expression of CD36 and lipid content; conversely, these effects disappeared after PRLR silencing. Furthermore, overexpression of CD36 significantly reduced the PRL-mediated improvement in lipid content (104).

## Future challenges

### HomeoFIT-PRL: Proposal of a new nosological entity

Given the evidence of PRL as a metabolic hormone, recently a new classification of PRL levels has been proposed, also introducing the novel concept of homeostatic functionally increased transient prolactinemia (HomeoFIT-PRL, 11). PRL levels ≤ 25 μg/L are currently considered normal, whereas the suspicion of prolactinoma arises when PRL exceeds 200 μg/L (3). Particularly, excluding the known causes of hyperprolactinemia as pregnancy, lactation, hypothyroidism, and drugs, PRL values within 25 -100 μg/L could be a response to temporary stimuli, like stress, insulin-induced hypoglycemia, sexual arousal, intensive exercise training, and circadian peaks, which requires to the subject a metabolic adaptation (11). Both in animals and humans, slightly high PRL levels (about 40 μg/L) have been associated with a lower prevalence of metabolic impairment. As consequence, it is possible to assume that PRL values ranging from 25 to 100 μg/L, in absence of other recognizable pathological causes, could represent a physiological response to the request of an increase in metabolic activity. For this reason, the denomination HomeoFIT-PRL has been proposed to define this range of PRL values. Further studies concerning the metabolic status of subjects with PRL levels within the HomeoFIT-PRL ranges are needed to better elucidate its impact on body weight, gluco-insulinemic and lipid profile. According to this evidence, the results of these studies could lead to the hypothesis of a less stringent PRL target in patients with prolactinoma treated with DAs in order to avoid the metabolic detrimental effect due to excessively low PRL levels.

#### DAs therapy in postmenopausal women with prolactinoma to improve cardiometabolic impairment

Although no peculiar studies regarding metabolism after menopause in patients with prolactinomas are currently available, the addition of hyperprolactinemia-related detrimental effect to the metabolic impairment ascribable to the decline in estrogens, physiologically occurring in this period, could be hypothesized in these patients. In healthy postmenopausal women, the unfavorable cardiometabolic impact of hyperprolactinemia has been recently demonstrated. Indeed, PRL has been identified as an independent predictor of central and peripheral blood pressure and arterial stiffness, being significantly correlated to HeartScore, a composite index able to predict 10-yr cardiovascular mortality, in healthy women during the first decade after menopause onset (105). Similarly, in healthy postmenopausal women changes in hemodynamic parameters and endothelial function have been reported to be predicted by PRL levels, even if normal (106). Based on these findings, in women with prolactinomas after menopause, besides the common higher risk of developing cardiovascular diseases as compared to fertile age, a further increase in cardiovascular risk might depend on the metabolic impact of PRL excess, thus suggesting a greater risk of a cardiovascular event in these patients as compared to healthy postmenopausal women. Considering the well-known DAs beneficial metabolic effects, DAs treatment in postmenopausal women could have a favorable impact on metabolic profile both directly and indirectly through the reduction of PRL levels (107). Currently, given the physiologic decline in PRL levels occurring with menopause, DAs treatment can be withdrawn in asymptomatic women with microprolactinoma considering that tumor enlargement rarely occurs (107). Conversely, in women with evidence of macroprolactinoma and concomitant signs and symptoms of mass effects, DAs should be continued independently from PRL levels to avoid any possible tumor enlargement (108). However, in light of the beneficial metabolic impact of DAs on body composition, MetS prevalence, gluco-insulinemic and lipid profile abnormalities, all being frequently seen in menopausal women as a consequence of estrogen deficiency, the decision to stop DAs after menopause should be tailored to the metabolic status and cardiovascular risk of individual patients (109–113). Future studies are required to clarify if therapy with DAs might be continued after menopause considering the potential beneficial impact on cardiometabolic health, regardless of PRL and tumor status.

## Conclusions

PRL exerts a wide spectrum of metabolic actions. Since both PRL excess and deficiency have been demonstrated to play a detrimental action on metabolism, PRL levels within the normal range appear to be essential to ensure metabolic homeostasis. For this reason, in patients with hyperprolactinaemia administered with DAs it might be useful to aim for PRL levels within the normal range to avoid a possible unfavorable metabolic effect due to excessively low PRL. Furthermore, the negative association between low PRL levels, obesity, gluco-insulinemic and lipid impairment have also been observed in non-hyperprolactinemic subjects, thus leading to the hypothesis that PRL investigation should be offered also to obese or diabetic patients, besides those with suspicion of hyperprolactinemia. Future studies will better clarify the role and the burden of PRL excess or deficiency on metabolic alterations, also investigating the potential role of PRL as a therapeutic target for non-hyperprolactinemic subjects suffering from metabolic diseases.

## Author contributions

RPir and RSA contributed to conception and design of the study. RPir and RSA wrote the first draft of the manuscript. GC prepared the figure and carried out the bibliographic research. AC and RPiv provided a significant expert contribution in the scientific content revision process and revised it for important intellectual content. RSA critically reviewed the manuscript. All authors contributed to the article and approved the submitted version.

## Conflict of interest

The authors declare that the research was conducted in the absence of any commercial or financial relationships that could be construed as a potential conflict of interest.

## Publisher’s note

All claims expressed in this article are solely those of the authors and do not necessarily represent those of their affiliated organizations, or those of the publisher, the editors and the reviewers. Any product that may be evaluated in this article, or claim that may be made by its manufacturer, is not guaranteed or endorsed by the publisher.
